# Oral Emergency Contraception Provision in the Veterans Health Administration: a Retrospective Cohort Study

**DOI:** 10.1007/s11606-022-07596-0

**Published:** 2022-08-30

**Authors:** Lori M. Gawron, Tao He, Lacey Lewis, Hannah Fudin, Lisa S. Callegari, David K. Turok, Vanessa Stevens

**Affiliations:** 1 Division of Family Planning, Department of Obstetrics and Gynecology, University of Utah, 30N 1900E Rm 2B-200, Salt Lake City, UT 84132 USA; 2 VA Salt Lake City Health Care System, Salt Lake City, UT USA; 3 Division of Epidemiology Department of Internal Medicine, University of Utah, Salt Lake City, UT USA; 4 VA Puget Sound Health Care System, Seattle, WA USA; 5 Department of Obstetrics and Gynecology, University of Washington, Seattle, WA USA

**Keywords:** Female veterans, Emergency contraception, unintended pregnancy

## Abstract

**Background:**

In the USA, oral emergency contraception (EC) use to prevent unintended pregnancy is increasing. Oral EC methods include levonorgestrel (LNG) and ulipristal acetate (UPA), with increased UPA efficacy over LNG in high BMI users and those beyond 3 days post intercourse. The Veterans Health Administration (VHA) provides oral EC at low or no cost, yet prescription-level Veteran data are lacking.

**Objective:**

To describe oral EC provision in VHA, including method type and Veteran user and prescriber characteristics.

**Design:**

A retrospective cohort study using VHA administrative data.

**Participants:**

All VHA oral EC prescriptions from January 1, 2016, to December 31, 2020.

**Main Measures:**

We linked Veteran-level sociodemographic and military characteristics and provider-level data with each prescription to identify variables associated with oral EC method.

**Key Results:**

A total of 4280 EC prescriptions (85% LNG) occurred for 3120 unique Veterans over 5 years. While prescriptions remained low annually, the proportion of UPA prescriptions increased from 12 to 19%. Compared to LNG users, UPA users were older (34% vs 25% over age 35 years, *p* <0.001); more likely to identify as white (57% vs 46%) and non-Hispanic (84% vs 79%) (*p* <0.001); and more likely to have a BMI ≥ 25 (76% vs 67%, *p* <0.001). UPA prescriptions originated most frequently from VA Medical Centers (87%) and women’s health clinics (76%) compared to community-based or other clinic types. In multivariable regression models, race, ethnicity, BMI ≥30, and prescriber facility type of a VA Medical Center or a women’s clinic location were predictive of UPA prescription.

**Conclusions:**

Oral EC provision in VHA remains low, but UPA use is increasing. LNG prescription occurs frequently in high BMI Veterans who would benefit from increased efficacy of UPA. Interventions to expand oral EC access in VHA are essential to ensure Veterans’ ability to avert unwanted pregnancies.

## INTRODUCTION

Access to a full range of contraceptive options is essential to support individuals in achieving their reproductive goals, as well as meeting the public health objective of decreasing unintended pregnancy in the USA.^1^ Oral emergency contraception (EC) can decrease the risk of unwanted pregnancy following an episode of unprotected intercourse. Data from the National Survey of Family Growth demonstrates that use of EC in the US has risen over the past 15 years, with 22% of reproductive age respondents reporting ever use of EC in 2015–2017 compared to only 4.2% in 2002.^2^

Oral EC methods include levonorgestrel (LNG), a synthetic progestogen, and ulipristal acetate (UPA), a progesterone receptor modulator.^3^ Oral LNG is most accessible in the USA, as it can be purchased over the counter (OTC), but is less effective compared to UPA in individuals with a body mass index (BMI) ≥25 and in those >72 h but within 5 days of intercourse.^4,5^ UPA requires a prescription and both methods may present financial barriers due to method cost or prescription co-pays. Additional barriers to use of either option include lack of pharmacy stock, lack of knowledge on difference between methods, and geographic variation in access.^6–8^ The National Survey of Family Growth data highlight rural-urban differences with 15% of rural respondents and 27% of urban respondents reporting oral EC use.^8^

Unlike civilian health systems, which often have cost or insurance barriers to accessing contraception, the Veterans Health Administration (VHA) provides a full range of contraceptive options for Veterans as part of their benefits. While oral EC cannot be purchased over the counter in VHA, both oral LNG and UPA can be accessed by prescription through the VHA pharmacy for a $9 co-pay or cost-free, depending on factors such as income or service-connected disability rating. Despite this VHA coverage, female Veterans experience unintended pregnancy at comparable rates to the general population.^9^ Previous VHA studies describe disparities in contraceptive use among Veterans related to race and ethnicity, race-based discrimination, and mental health or substance use disorders.^10–12^ While these previous studies describe hormonal contraceptive methods with ongoing use, such as pills, patches, or rings, data on the episodic, single-dose, oral EC provision in the VHA are lacking. As barriers to oral EC provision in the VHA may be different from civilian health systems and disparities in vulnerable VHA populations may exist, further study is warranted to ensure female Veterans can access oral EC in a timely fashion when needed. We also hypothesize that despite both LNG and UPA requiring a prescription in the VHA, use of UPA will be more limited, due to local formulary restrictions and lack of provider awareness of the option or knowledge regarding efficacy differences. Thus, the objectives of this study are to describe VHA oral EC provision, including type of oral EC, and to compare Veteran and prescribing provider characteristics by EC method.

## METHODS

This is a retrospective cohort study using VHA clinical and administrative data. The University of Utah Institutional Review Board in conjunction with Salt Lake City VA Research and Development approved this study protocol. We used a nationwide VHA research database of administrative and clinical data from VHA Corporate Data Warehouse (CDW) managed by the Veteran’s Informatics and Computing Infrastructure (VINCI).^13^ We identified all outpatient EC prescriptions for oral LNG and UPA and date of fill in the VHA from January 1, 2016, to December 31, 2020. We linked each prescription with Veteran and provider characteristics from clinical and administrative tables in VINCI. Each prescription was a unique “participant” and Veterans may have more than one prescription in the dataset. Oral EC is for an episode of unprotected intercourse and new prescriptions or refills are for recurrent unique episodes with potentially different Veteran (e.g., age, BMI) or provider (e.g., clinical location) characteristics related to the fill or different oral EC type used by the same Veteran.

Veteran characteristics included age at time of prescription fill, sociodemographic variables, BMI (defined as a height from any episode and the closest recorded weight in the clinical records to the prescription fill), and military characteristics, including a report of military sexual trauma history (MST), as it has been associated with an increase in contraceptive use^14^. Provider characteristics included provider type, including physician, advanced practice nurse or physician assistant, pharmacist, and “unknown,” which includes non-VHA prescribers when a Veteran fills the prescription in a VHA pharmacy. Type of practice setting included VA medical centers (VAMC), which are the referral hub for a catchment area with inpatient and outpatient services, community-based outpatient clinic (CBOC), which tends to have primary care outpatient services only, and “unknown,” which includes telehealth or virtual care locations or non-VHA locations from providers who write prescriptions for in-VHA fills. Clinic type included gynecology, general primary care, women’s health primary care, mental health, and emergency department or urgent care clinics.

We compared Veteran and provider descriptive characteristics associated with each prescription fill by type of oral EC (LNG vs UPA). We reported counts of prescriptions by type and calendar year (2016–2020) and calculated proportion of UPA vs LNG each year. We explored Veteran and provider characteristics associated with oral EC type via multivariable logistic regression models fit within the generalized estimating equations framework, accounting for clustering of prescriptions within patients and of patients within facility (repeated measures). Variables were included in the model based on clinical factors that may influence the likelihood of receiving UPA (e.g., BMI^4^) or prior literature on contraceptive users^15^ and we retained variables in the model regardless of statistical significance. We handled missing values by including a separate category for missing information to include as many patients as possible in the model.

## RESULTS

A total of 4280 oral EC prescriptions occurred for 3120 unique Veterans over the 5-year study timeframe and, of these, 85% were LNG. UPA prescriptions increased from an average of 104 per year in the first 3 years (12%) to an average of 167 per year in 2019 to 2020 (19%). UPA users were older than LNG users with mean age of 33 years vs 32 years and with 34% vs 25% over age 35 years (*p* <0.001) (Table 1). Compared to LNG users, a greater proportion of UPA users identified as white (57% vs 46%; *p* <0.001) and non-Hispanic (84% vs 79%; *p* <0.001), and had a higher mean BMI (33 vs 32; *p* <0.001). We found 76% of UPA users and 67% of LNG users had a BMI ≥25 (*p* <0.001). Nearly all (98%) weight measurements identified were within 1 year of the prescription date. The majority of prescribers were physicians and there was no significant difference in UPA versus LNG prescriptions by provider type (*p*=0.027). Only 62 UPA prescriptions (9.6%) originated in a CBOC over 5 years, while 563 originated from a VA Medical Center. In multivariable regression models, Veteran characteristics of white race (OR 2.10; 95%CI 1.45, 3.03), non-Hispanic ethnicity (OR 1.72; 95%CI 1.15, 2.57), and BMI ≥30 (OR 2.13; 95%CI 1.55, 2.94) were predictive of UPA prescription. Additionally, the prescriber facility type of a VA Medical Center (OR 2.71; 95%CI 1.59, 4.64) or a women’s (primary care or gynecology) clinic location (OR 2.59; 95%CI 1.74, 3.86) were also predictive of UPA prescription (Table 2).

**Table 1:** Veteran and Provider Characteristics Associated with Oral Emergency Contraception Prescriptions in the Veterans Health Administration from 2016 to 2020 by Method Type (Prescription *N*=4280)

Variable	Oral LNG	UPA	*p*-value
Total prescriptions	3635	645	
Unique Veterans	2641	479	
No. prescriptions per Veteran			0.012
(median [IQR])	1.0 [1.0, 1.0]	1.0 [1.0, 2.0]	
No. prescriptions per facility			
(median [IQR])	15.0 [5.5, 35.0]	4.5 [2.0, 13.3]	<0.001
Prescriptions by year (%)			<0.001
2016	734 (20.2)	102 (15.8)	
2017	723 (19.9)	101 (15.7)	
2018	661 (18.2)	109 (16.9)	
2019	806 (22.2)	173 (26.8)	
2020	711 (19.6)	160 (24.8)	
Age (mean (SD)	31.98 (6.06)	33.13 (6.13)	<0.001
Age group (%)			<0.001
18 – 34	2729 (75.1)	423 (65.6)	
35 – 44	789 (21.7)	198 (30.7)	
45+	117 (3.2)	24 (3.7)	
Race			<0.001
Black	1417 (39.0)	188 (29.1)	
White	1655 (45.5)	367 (56.9)	
Other	254 (7.0)	43 (6.7)	
Missing/unknown	309 (8.5)	47 (7.3)	
Ethnicity			0.001
Hispanic or Latino	593 (16.3)	69 (10.7)	
Non-Hispanic or Latino	2855 (78.5)	543 (84.2)	
Missing/unknown	187 (5.1)	33 (5.1)	
Marital status			0.104
Divorced/separated/widowed	1203 (33.1)	189 (29.3)	
Married	745 (20.5)	134 (20.8)	
Single	1652 (45.4)	311 (48.2)	
Missing/declined	35 (1.0)	11 (1.7)	
Body mass index (mean (SD))	28.21 (6.1)	29.9 (6.1)	<0.001
Body mass index (%)			<0.001
<25	1168 (32.1)	152 (23.6)	
25 – 29.9	1217 (33.5)	185 (28.7)	
≥30	1198 (33.0)	302 (46.8)	
Missing/unknown	52 (1.4)	<10 (<1.0)	
Percent service connected disability (%)			0.352
0 – 50	747 (20.6)	129 (20.0)	
51–100	2428 (66.8)	421 (65.3)	
Missing/unknown	460 (12.7)	95 (14.7)	
Branch of service			<0.001
Air Force	560 (15.4)	152 (23.6)	
Army	1644 (45.2)	266 (41.2)	
Marine Core	392 (10.8)	43 (6.7)	
Navy	971 (26.7)	163 (25.3)	
Other	43 (1.2)	18 (2.8)	
Unknown/missing	25 (0.7)	<10 (<1.0)	
Military sexual trauma			0.184
Yes	1448 (42.6)	256 (39.7)	
Combat service			<0.001
No	1880 (51.7)	345 (53.5)	
Yes	347 (9.5)	23 (3.6)	
Missing/unknown	1408 (38.7)	277 (42.9)	
Facility type			<0.001
CBOC	838 (23.1)	62 (9.6)	
VAMC	2558 (70.4)	563 (87.3)	
Other/unknown	239 (6.6)	20 (3.1)	
Clinic type			
Women’s health primary care	1232 (33.9)	361 (56.0)	<0.001
General primary care	1032 (28.4)	82 (12.7)	<0.001
Gynecology	655 (18.0)	127 (19.7)	0.339
Mental health	42 (1.2)	<10 (<1.0)	1.000
Emergency department	178 (4.9)	<10 (<1.0)	<0.001
Pharmacy	114 (3.1)	17 (2.6)	0.578
Provider type			0.027
APN or PA	1117 (30.7)	171 (26.5)	
Pharmacist	144 (4.0)	16 (2.5)	
Physician	2362 (65.0)	456 (70.7)	
Other/unknown	12 (0.3)	2 (0.3)	

*LNG*, levonorgestrel; *UPA*, ulipristal acetate; *SD*, standard deviation; *CBOC*, community-based outpatient clinic; *VAMC*, Veteran Administration Medical Center; *APN*, advance practice nurse; *PA*, physician assistant

**Table 2:** Predictors of Ulipristal Acetate Prescription in the Veterans Health Administration from 2016 to 2020

Variable	(OR, 95% CI)
Year	
2016	Ref
2017	1.07 (0.57, 2.01)
2018	1.27 (0.74, 2.19)
2019	1.62 (1.00, 2.62)
2020	1.58 (0.92, 2.71)
Veteran age, per year	1.02 (1.00, 1.04)
Race	
Black	Ref
White	2.10 (1.45, 3.03)
Other	1.33 (0.73, 2.44)
Missing/unknown	1.54 (0.93, 2.53)
Hispanic ethnicity	
Yes	Ref
No	1.72 (1.15, 2.57)
Missing/unknown	1.64 (0.90, 2.99)
Marital status	
Declined/missing	Ref
Divorced/separated/widow	0.57 (0.24, 1.36)
Married	0.65 (0.26, 1.61)
Single	0.70 (0.29, 1.68)
Body mass index	
<25	Ref
25–29.9	1.25 (0.91, 1.70)
≥30	2.13 (1.55, 2.94)
Unknown/missing	0.88 (0.29, 2.74)
Branch of service	
Air Force	Ref
Army	0.67 (0.42, 1.06)
Marine Core	0.52 (0.31, 0.88)
Navy	0.72 (0.46, 1.14)
Other	1.48 (0.65, 3.37)
Unknown/missing	0.38 (0.08, 1.77)
Percent service connected disability (%)	
0–50	Ref
51–100	1.01 (0.69, 1.27)
Missing/unknown	1.10 (0.76, 1.35)
Military sexual trauma	
No	Ref
Yes	0.90 (0.69, 1.17)
Combat service	
No	Ref
Yes	0.41 (0.21, 0.80)
Facility type	
CBOC	Ref
VAMC	2.71 (1.59, 4.64)
Other/unknown	2.38 (0.99, 5.69)
Clinic type	
Non-WH or GYN	Ref
WH primary care or GYN	2.59 (1.74, 3.86)
Provider type	
APN or PA	Ref
Pharmacist	1.08 (0.35, 3.34)
Physician	1.25 (0.66, 2.36)
Unknown	1.55 (0.16, 14.92)

*OR*, odds ratio; *CI*, confidence interval; *Ref*, reference; *CBOC*, community-based outpatient clinic; *VAMC*, Veterans Affairs Medical Center; *GYN*, gynecology; *WH*, women’s health; *APN*, advance practice nurse; *PA*, physician assistant

## DISCUSSION

We describe national oral EC provision in the VHA over a 5-year timeframe, which remains extremely low, despite lower cost compared to over the counter oral LNG EC in non-VHA pharmacies. Using the most recent estimate of 189,000 women Veterans aged 18–44 years in the VHA in fiscal year 2015 and known increases in enrollees since then^16^, less than 0.5% received oral EC in any of the years of analysis. We lack a civilian data comparator of annual use of oral EC. However, based on 22% of the general population reporting ever use of EC and similarities between Veteran and civilian contraceptive use demonstrated elsewhere, we anticipated higher numbers of VHA EC prescriptions than we observed.^2,15^

National civilian data show 11% of non-Hispanic white survey respondents and 11% of Hispanic respondents reporting ever use of oral EC compared to only 8% of non-Hispanic black respondents. With 42% of women Veterans identifying as a racial or ethnic minority in fiscal year 2015^16^, this study highlights an opportunity to address the racial and ethnic differences we found in VHA oral EC provision in this study. The low provision of oral EC in the VHA could be related to lack of awareness by Veterans and/or providers of this pharmacy option, provider biases in counseling or prescription, Veteran desire for ease of OTC provision rather than asking for VHA pharmacy dispensing, or provider or health system barriers that do not allow for timely access within 5 days of unprotected intercourse. As 40% of the two million total women Veterans and 98% of the two hundred thousand active-duty service women are of reproductive age^16^, understanding and addressing barriers to EC provision is timely, while anticipating continued future growth in VHA enrollment.

The low provision of UPA is concerning as 67% of the oral LNG users had a BMI ≥25, and efficacy of LNG among individuals with elevated BMI is lower compared to UPA.^4^ A national initiative to ensure UPA is on local VHA and non-VHA contracted pharmacy formularies would be the first step to ensure universal access to both oral EC options, as currently, formularies often carry only one EC option, with oral LNG as the default. Data on efficacy differences between UPA and LNG have been published in the past decade, thus dissemination and education of prescribers who have practiced for many years or may not care for many female Veterans will need to be prioritized across the VHA. Each VHA pharmacy may also have differing or outdated policies that result in a barrier to UPA prescription, such as a requirement for pre-prescribing pregnancy testing or an in-person visit, despite lack of evidence for these practices. With education and UPA access, VHA women’s health primary care providers can serve as champions in their settings, particularly in lower volume CBOCs.

Previous non-VHA interventions to improve timely access and overcome known access barriers include advanced supply of an oral EC dose^17^, virtual or internet-based contraceptive services^18^, and pharmacist education with direct outpatient pharmacist prescription^19^. The VHA is an ideal health system to incorporate all of these interventions due to Veteran pharmacy benefits, established telehealth services, and ambulatory pharmacists embedded into women Veteran’s primary care clinics who have prescribing authority, as well as outpatient pharmacists who could prescribe under a standing order. As female Veterans remain a minority in the VHA, another unique opportunity to expand oral EC and promote reproductive control and family planning within relationships is advance provision of oral LNG EC to male Veterans. Unlike UPA, oral LNG is sold in retail pharmacies as an OTC medication and does not need direct prescription to the user. The need for male EC provision, as well as acknowledgement of the male role in unintended pregnancy, have been established in a non-VHA clinic setting.^20^ Attention to family planning needs of male Veterans is understudied and ripe for interventions to improve care.

Limitations of this study include use of structured, retrospective data with resultant missing data for some variables. There is risk of misclassification of clinic types as some CBOCs across the health system with a higher volume of female Veterans offer expansive services that are similar to VA medical centers. Knowledge on the overall and location-specific denominator of female Veterans at risk of unintended pregnancy and in need of oral EC is lacking. As Veterans can request oral EC without an in-person visit, BMI measurements may be from an in-person visit separate from the EC prescription date and weight may change over time, although the majority were within 1 year. Some Veterans who seek care in CBOCs and need a time-sensitive medication such as oral EC are able to use their VHA pharmacy benefits through a non-VHA pharmacy program in their community and those prescriptions would not be captured in this dataset. Similarly, an OTC purchase of oral LNG for EC without using VHA pharmacy benefits would not be identified. This study focused solely on oral EC, but some Veterans may choose an intrauterine device insertion for EC, if accessible.

In conclusion, oral EC provision in VHA remains low, but the proportion of UPA prescriptions has increased in recent years. Opportunities to ensure Veterans receive the most effective EC option exist, particularly in those with a higher BMI, and interventions to expand oral EC access in VHA are essential to support Veterans in their reproductive autonomy and goals.
